# Serum adiponectin and cortisol levels are not affected by studied *ADIPOQ* gene variants: Tehran lipid and glucose study

**DOI:** 10.1186/s12902-022-01020-8

**Published:** 2022-04-18

**Authors:** Masoumeh Nezhadali, Seyed Alireza Mesbah-Namin, Mehdi Hedayati, Mahdi Akbarzadeh, Leila Najd Hassan Bonab, Maryam S. Daneshpour

**Affiliations:** 1 Department of Biology, Islamshahr Branch, Islamic Azad University, Islamshahr, Iran; 2 Department of Clinical Biochemistry, Faculty of Medical Sciences, Tarbiat Modares University, Tehran, Iran; 3 Cellular and Molecular Endocrine Research Center, Research Institute for Endocrine Science, Shahid Beheshti University of Medical Science, Tehran, Iran

**Keywords:** *ADIPOQ*, Obesity, Cortisol, Adiponectin

## Abstract

**Background:**

Obesity is a major public health concern in developed and even developing countries worldwide. Adiponectin is a protein secreted by adipose tissue that modulates many metabolic processes and plays a vital role in obesity. This study aimed to determine the association of four variants of the *ADIPOQ* gene with serum adiponectin, cortisol levels and obesity status.

**Methods:**

This case-control study was performed on 164 obese individuals compared by 156 control from the Tehran Lipid and Glucose Study (TLGS). Standard procedures obtained anthropometric measures and metabolic parameters. Cortisol and adiponectin levels were measured *by* ELISA method. rs1501299, rs266729, rs17300539, and rs17366743 on the *ADIPOQ* gene were genotyped using the PCR-RFLP. The correlation between adiponectin gene SNPs and obesity were calculated by Additive, dominant, and recessive genetic models. Pearson’s or Spearman’s found correlations between adiponectin levels and metabolic and anthropometric variables. Data were analyzed using SPSS software Version 20.

**Results:**

Adiponectin and cortisol levels were significantly lower in obese subjects compared to the control group (*p* < 0.05). There was a significant negative correlation between serum adiponectin level and BMI, waist circumference (WC), waist-hip ratio, hip circumference (HC), Fasting blood sugar (FBS) Triglyceride (TG), Total cholesterol (TC), Systolic blood pressure (SBP), Diastolic blood pressure (DBP) (*r* = − 0.147, *r* = − 0.324, *r* = 0.371, *r* = − 0.179, *r* = − 0.299, *r* = − 0.277, *r* = − 0.041, *r* = − 0.134, and *r* = − 0.149, respectively). A positive correlation was found between adiponectin and high-density lipoprotein cholesterol (HDL-C) (*r* = 0.29), but no significant correlations were found between adiponectin and Low-density lipoprotein cholesterol(LDL-C) and cortisol. *ADIPOQ* variant rs1501299 was significantly associated with cortisol levels in subjects with BMI ≥ 25 (*P*-value =0.039).

**Conclusions:**

Adiponectin and cortisol levels were associated with obesity. No *ADIPOQ* gene variants and haplotypes were associated with cortisol, Adiponectin, and obesity.

## Background

The prevalence of obesity has increased significantly in recent years, and if the current trend continues, it is predicted that by 2030, more than 58% of adults worldwide will be overweight or obese [1]. Obesity can disrupt well-being and lead to death [2]. Excess body fat is stored in adipose tissue, but adipocytes also have functions other than simple storage cells. The most important of these is the capacity for protein secretion*.* These peptides are called adipocytokines or adipokines. Adipokines seem to have functioned as modulators of metabolism. Adiponectin is one of the most abundantly secreted adipokines [3] and in obesity it becomes dysregulated [1]. Adiponectin is produced by subcutaneous and visceral fat [4] and has anti-inflammatory properties; therefore, it is associated with metabolic disorders such as obesity, type II diabetes, coronary heart disease, and metabolic syndrome [5].

Adiponectin levels can be affected by age, sex, and body mass index [6, 7]. In lean individuals, Adiponectin induces uptake of fatty acids and enhances adipocyte lipid storage*,* and when the adipose cells reach a certain size, it is not secreted to prevent further lipid accumulation [3]. In other words, adiponectin levels decrease in obese individuals [8]. Notably, its mechanism has not yet been well understood, but higher levels of TNF-a or products secreted by visceral adipose tissue in obese people may result from the inhibition of adiponectin synthesis or secretion [9].

Adiponectin is a novel protein of 30 kDa [10] that consists of 244 amino acid [8]. Adiponectin comprises three forms (trimer, hexamer, and high-molecular-weight multimers) with different biological activities [9].

Adiponectin activates AMP-dependent kinase (AMPK) and AMP kinase activation enhances glucose uptake and reduces Gluconeogenesis triggering fatty acid oxidation [3, 11]. Thus, plasma adiponectin levels are inversely correlated with obesity, hypertension, triglyceride, total cholesterol, and LDL-cholesterol levels [12]. In obesity, the imbalance between 11β-reductase and 11β-dehydrogenase activities likely promotes cortisol accumulation in adipose and leads to adverse metabolic consequences. Findings have shown increased cortisol regeneration within adipose tissue in obesity [13] and an inverse relationship between cortisol and adiponectin concentrations [14].

Plasma adiponectin concentrations are heritable [15] and associated with genetic variation in the adiponectin gene [16]. Approximately 40-70% of the change in plasma adiponectin levels is affected by genetic variants [12]. Adipose most abundant gene transcript 1(APM1) is the best candidate for regulating adiponectin Levels [12, 15]. Although APM1 variants’ role in the development of obesity is widely recognized, their clear contribution is still not fully understood. There are conflicting findings in association studies due to differences in age, genetic or ethnic background of study populations. The *ADIPOQ* gene is located in region 3q27 of the chromosome and consists of three exons and two introns [6] recognized as a susceptible locus for type 2 diabetes and obesity [17]. Two promoter single nucleotide polymorphisms (rs17300539 and rs266729) at the adiponectin gene are associated with altered plasma adiponectin concentration and obesity [15]. The SNP, rs1501299 (276G/T) in intron two, is associated with obesity and MetS [18]. Another SNP rs17366743 (Y111H) is located in exon 3 of *ADIPOQ*, which changes the T allele into the C allele [19]. The present study evaluated the association of single nucleotide polymorphisms (rs1501299, rs266729, rs17300539, and rs17366743) and haplotypes in the Adiponectin (*ADIPOQ*) gene, with serum adiponectin and cortisol levels, and obesity.

## Methods

### Study design

The subjects for this study were selected from the Tehran Lipid and Glucose Study (TLGS, a large-scale prospective, community-based study conducted on 15,005 subjects to determine the prevalence of non-communicable disease risk factors) [20] and the details have been published earlier. The Research Institute for Endocrine Sciences’ research ethics committee, Shahid Beheshti University of Medical Sciences, approved the study protocol. Written informed consent was obtained from all the participants.

### **Anthropometric and biochemical measurements**

Anthropometric parameters (height, weight, waist circumference, waist to hip ratio) and blood pressure were evaluated using standard methods. The weight was measured without shoes, in a standing position, using a calibrated balance (in Kilograms). The height was also measured using a constant tape measure (in centimeters). BMI was calculated as [weight (kg) /height (m^2^)]. Also, factors associated with obesity, including body mass index and blood pressure, were measured. Two peripheral blood samples from participants were obtained, one without serum anticoagulant for clinical measurements and the other containing anticoagulant (containing EDTA 3 mg/ml) for DNA extraction. Blood serum samples were used for the analysis of biochemical factors. The levels of cholesterol, triglyceride, −HDL-C, and fasting blood glucose were measured using the enzymatic colorimetric method (Pars Azmoon Company). In addition, the measure of LDL-C was calculated by the Freidwald formula (CHOL-HDL-TG/5 = LDL (mg/dl) [21]. The adiponectin and cortisol hormones measurements were performed using a commercial human Elisa Kit (Mercodia Company, Sweden). Adiponectin and cortisol levels were determined by sandwich ELISA kit through two antigen-specific antibodies, a purified immunoglobulin bound to a solid phase, and one enzyme-linked detection antibody, and then the concentration of adiponectin and cortisol of the samples was measured spectrophotometrically at 450 nm in a microplate reader.

Initially, all Tehran Lipid and Glucose Study participants were divided into two groups based on body mass index. In this classification, people with a BMI of less than 25 kg/m^2^ were considered normal-weight individuals, and people with BMI greater than 25 kg/m ^2^ were in the obese group [22]. The target population inclusion criteria were over 18 years of age, triglycerides < 400 mg/dL, not taking medication, absence of cardiovascular disease, and hypertension. We also excluded individuals with a history of significant hepatic, renal, thyroid dysfunction, recent surgical operations, history of cardiovascular diseases, and pregnancy. Based on the mentioned criteria, 320 subjects with a mean age of 44 ± 14, including 156 males and 164 females, were randomly selected from the Tehran Lipid and Glucose Study.

### Genotyping

For the present study, four single nucleotide polymorphisms (SNPs) in the adiponectin gene, *ADIPOQ* were selected based on the previously associated markers with obesity. Genomic DNA was extracted from peripheral blood using the standard salting-out method and stored at − 20 °C [23]. The four SNPs in the *ADIPOQ* gene were genotyped using polymerase chain reaction-restriction fragment length polymorphism (PCR-RFLP). The initial primer pairs were designed using the software Gene runner, and then Blast primers, and there were finally obtained from CinnaGen. The PCR reaction mixture (25 μl) contained one λ genomic DNA (50-100 ng/μl), dNTPs, Taq DNA Polymerase, MgCl_2_ and primer pairs (Cinaagen Co., Tehran, Iran), 0.9 μl forward primer, and 0.9 μl reverse primers (10 pm/μL). PCR was performed under the following conditions: initial denaturation, 5 min at 94 °C, then 40 cycles and every cycle of denaturation at 95 ͦ C for 30 s, annealing at 64 °C for 45 s for rs17300539 and rs266729, annealing at 61 ͦC for 30 s for SNP rs17366743 and extension at 72 ͦC for 45 s. For rs1501299, 10 min at 93 °C, followed by 35 cycles of 45 s at 93 °C, 30 s at 58 °C, 45 s at 72 °C. The final extension was performed at 72 °C for 5 min. SNPs rs17300539 and rs266729 of the adiponectin gene promoter are located close in *ADIPOQ* so a pair of primers were amplified for two SNPs. The sequence of the primers used in PCR with the corresponding PCR product sizes *ADIPOQ* gene SNPs are shown in Table 1. The validity of PCR and the DNA fragments were electrophoresed on a 2% agarose gel. In this stage, PCR products were kept for 16 h with a restriction enzyme and incubated in 37 °C. All the restriction enzymes were purchased from Fermentas Company. The product size of the PCR and the RFLP fragments of SNPs of the *ADIPOQ* gene are shown in Table 1. All the restriction enzymes were purchased from Fermentas Company. For determining the genotypes, 2% agarose gel electrophoresis was used.

**Table 1:** The primers and PCR product size for 4 SNPs in the ADIPOQ gene

ID	Major/minor allele	Primer (sequence 5′ to 3′)	Restrection Enzyme	PCR Restrection	RFLP fragments (bp)
rs17300539	G/A	F: 5′-AGGCTCTGTGTGGACTGTGGA-3′ R: 5′-CCTGGAGAACTGGAAGCTGC-3′	MspI	296	AA = 296 GA = 129,167,296 GG = 129,167
rs266729	G/C	F: 5′-AGGCTCTGTGTGGACTGTGGA-3′ R: 5′-CCTGGAGAACTGGAAGCTGC-3’	HhaI	296	CC = 296 GC = 113,183,296 GG = 113,183
rs1501299	G/T	F: 5’- GGCCTCTTTCATCACAGACC-3′ R: 5′-AGATGCAGCAAAGCCAAAGT-3’	BsmI	196	TT = 196 GT = 50,146,196 GG = 50,146
rs17366743	T/C	F:5’-TAAGGGAGACATCGGTGAAAC-3′ R: 5′-TTACGCTCCTTCCCCATA −3’	Bst1107	438	CC = 438 TC = 104.334,438 TT = 104.334

### Data management and statistical analysis

Normally distributed continuous variables were expressed as mean ± SD, and skewed continuous variables were expressed as the median and interquartile range (IQR 25-75%). Categorical variables were also reported as frequency. Three distinguished models consisting of additive, dominant, and recessive models were utilized to compare genotype and allele frequencies between two groups (nonobese and obese), all of which were adjusted by gender and age. The genotype frequency distributions of four SNPs were in concurrence with Hardy–Weinberg Equilibrium (Pearson’s Chi-square statistic test). Normally distributed variables were analyzed using a two-tailed independent sample t-test, while variables with a skewed distribution were analyzed using mann Whitney. Pearson correlation was used for testing the correlation between continuous variables. Also, the Spearman correlation coefficient assessed associations of adiponectin level with the skewed or normal distribution of anthropometric or metabolic characteristics. The differences with *P*-values less than 0·05 were considered significant. All the analyses were performed using SPSS, version 20 (SPSS, Chicago, IL, USA).

## Results

Serum total adiponectin levels ranged from 2.3 μg/ml to 24.8 μg/ml. The serum concentrations of total adiponectin levels in women (3.00–24.80 mg/l) were significantly higher (*p* = 0.006) than those in men (2.30–17.00 mg/l). Anthropometric and biochemical features of the studied population are illustrated in Table 2. The obese group exhibited a significantly higher BMI, HC, LDL-C, FBS, TG, SBP, DBP than the control group (*P*-value < 0.05), but cortisol was significantly lower in the obese group. The results showed that the mean serum adiponectin was 6.2 μg/ml in the obese group (BMI ≥ 25) compared to in the control group, 7.8 μg/ml (BMI < 25) (*p* = 0.015). There were no significant differences in age and HDL-C between obese and normal participants (Table 2).

**Table 2:** Clinical characteristics of the study population

	Total	Non-obese	Obese	***P***-value
	(***n*** = 320)	(***n*** = 156)	(***n*** = 164)	
Age (year)	43 ± 13	43 ± 15	44 ± 11	0.38
Body mass index (kg/m^2^)	25 ± 5	20 ± 2	30 ± 3	< 0.001
Weight (kg)	68 ± 14	57 ± 8	78 ± 11	< 0.001
Height (cm)	163 ± 9	165 ± 9	161 ± 9	< 0.001
Hip circumference (cm)	98 ± 10	90 ± 5	106 ± 8	< 0.001
Waist circumference (cm)	87 ± 13	76 ± 8	97 ± 9	< 0.001
Waist to hip	0.89 ± 0.08	0.81 ± 0.007	0.91 ± 0.004	< 0.001
Triglyceride (mg/dL)	140 ± 72	113 ± 61	165 ± 72	< 0.001
Total cholesterol (mg/dl)	188 ± 41	174 ± 39	201 ± 39	< 0.001
High density lipoprotein cholesterol (mg/dl)	39 ± 10	40 ± 9	39 ± 10	0.15
Low density lipoprotein cholesterol (mg/dl)	120 ± 33	111 ± 35	128 ± 34	< 0.001
Systolic blood pressure (mm Hg)	113 ± 13	109 ± 13	117 ± 12	< 0.001
Diastolic blood pressure (mm Hg)	72 ± 8	70 ± 9	75 ± 7	< 0.001
Cortisol	11 ± 61	13 ± 7	10 ± 6	0.002
Adiponectin	6.5 ± 58	7.8 ± 58	6.2 ± 57	0.015
FBS (mg/dL)	91 ± 22	87 ± 15	96 ± 27	< 0.001

Difference between mean level, were analyzed by ANOVA for normally distributed variables and kruskal-Wallis test for variables with a skewed distribution

There was no evidence of any deviation from the Hardy–Weinberg equilibrium for *ADIPOQ* SNPs rs1501299, rs266729, rs17300539, and rs17366743. Table 3 shows the allelic and genotypic frequencies in subjects BMI ≥ 25 and control group BMI < 25, respectively, and also shows the association *ADIPOQ* SNPs with BMI in three genetic models and under two models (Model 1 after adjusting for age and sex and in model 2 after additionally adjusted for adiponectin and cortisol levels). The genotype frequencies distribution of SNPs of the *ADIPOQ* gene was not different between subjects with BMI ≥ 25 and the control group BMI < 25 under the recessive, dominant, and additive models. Therefore, no significant association was found between any SNPs of *ADIPOQ* with BMI under the three genetic models (Table 3).

**Table 3:** Associations of SNPs with BMI in three genetic models

SNP	Genetic Model	Genotype	Non-obese	Obese	Crude	Model 1	Model 2
			(***n*** = 156)	(***n*** = 164)	OR (CI- 95%) (***P***.value)	OR (CI- 95%) (***P***.value)	OR (CI- 95%) (***P***.value)
**bsm.rs1 SNP**	**Additive**	GG	89 (57.1)	88 (53/8)	1(references)	1(references)	1(references)
		GT	53 (34)	63 (38.4)	1.04 (0.75-1.92)	1.21 (0.76-1.97)	1.13 (0.68-1.87)
					*p* = 0.44	*p* = 0.41	*p* = 0.62
		TT	14 (9)	13 (7.9)	0.93 (0.41-2.12)	0.95 (0.41-2.17)	0.94 (0.39-2.25)
					*p* = 0.87	*p* = 0.90	*p* = 0.89
	**Dominant**	GG	89 (57.1)	88 (53.7)	1(references)	1(references)	1(references)
		GT/TT	67 (42.9)	76 (46.3)	1.14 (0.73-1.78)	1.16 (0.74-1.82)	1.09 (0.68-1.75)
					*p* = 0.94	*p* = 0.51	*p* = 0.70
	**Recessive**	GT/GG	142 (91)	151 (92.1)	1(references)	1(references)	1(references)
		TT	14 (9)	13 (7.9)	0.87 (0.39-1.92)	0.87 (0.39-1.97)	0.89 (0.38-2.09)
					*p* = 0.73	*p* = 0.74	*p* = 0.79
	**Alleles**	G	231 (74.1)	239 (72.8)	–	–	–
		T	75 (25.7)	78 (26)	–	–	–
**Mspl.rs2 SNP**	**Additive**	GG	134 (85.9)	146 (89)	1(references)	1(references)	1(references)
		GA	21 (13.9)	18 (11)	0.78 (0.41-1.50)	0.78 (0.39-1.54)	0.84 (0.42-1.78)
					0.48	*p* = 0.47	*p* = 0.66
		AA	1 (0.6)	0	1 (0.0001)	1 (0.0001)	1 (0.0001)
					*p* = 1	*p* = 1	*p* = 1
	**Dominant**	GG	134 (85.9)	146 (89)	1(references)	1(references)	1(references)
		GA/AA	22 (14.1)	18 (11)	0.75 (0.39-1.46)	0.74 (0.37-1.45)	0.79 (0.37-1.65)
					*p* = 0.39	*p* = 0.38	*p* = 0.53
	**Recessive**	AA	1 (0.6)	0	1(references)	1(references)	1(references)
		GA/GG	155 (99.4)	164 (100)	1 (0.0001)	1 (0.0001)	1 (0.0001)
					*p* = 1	*p* = 1	*p* = 1
	**Alleles**	G	289 (92.6)	310 (94.5)	–	–	–
		A	23 (7.7)	18 (5.5)	–	–	–
**Hha.rs3 SNP**	**Additive**	CC	93 (62)	102 (62.2)	1(references)	1(references)	1(references)
		CG	49 (32.7)	50 (30.5)	0.93 (0.57-1.50)	0.97 (0.59-1.59)	1.04 (0.61-1.77)
					*p* = 0.79	*p* = 0.92	*p* = 0.86
		GG	8 (5.3)	12 (7.3)	1.43 (0.55-3.67)	1.32 (0.51-3.42)	1.63 (0.60-4.45)
					*p* = 0.45	*p* = 0.56	*p* = 0.33
	**Dominant**	CC	93 (62)	102 (62.2)	1(references)	1(references)	1(references)
		CG/GG	57 (38)	62 (37.8)	1 (0.62-1.61)	1.02 (0.64-1.63)	1.12 (0.68-1.85)
					*p* = 0.99	*p* = 0.90	*p* = 0.39
	**Recessive**	CG/CC	142 (94.7)	152 (92.7)	1(references)	1(references)	1(references)
		GG	8 (5.3)	12 (7.3)	1.46 (0.58-3.70)	1.40 (0.55-3.59)	1.60 (0.60-4.3)
					*p* = 0.47	*p* = 0.48	*p* = 0.34
	**Alleles**	C	235 (78.3)	254 (77.4)	–	–	–
		G	65 (21.7)	74 (22.6)	–	–	–
**BST1107.rs4 SNP**	**Additive**	**TT**	149 (55.5)	157 (95.2)	1(references)	1(references)	1(references)
		CT	6 (3.9)	8 (4.8)	1.25 (0.42-3.70)	1.43 (0.47-4.30)	2.18 (0.69-6.45)
					*p* = 0.67	*p* = 0.52	*p* = 0.18
		CC	1 (0.6)	0	0.0001 (0.0001)	0.0001 (0.0001)	0.0001 (0.0001)
					*p* = 1	*p* = 1	*p* = 1
	**Dominant**	**TT**	149 (95.5)	156 (95.1)	1(references)	1(references)	1(references)
		CT/CC	7 (4.5)	8 (4.9)	1.12 (0.39-3.18)	1.23 (0.42-3.56)	1.82 (0.61-5.43)
					*p* = 0.82	*p* = 0.69	*p* = 0.28
	**Recessive**	**TT/CT**	155 (99.4)	164 (100)	1(references)	1(references)	1(references)
		CC	1 (0.6)	0	0.0001 (0.0001)	0.0001 (0.0001)	0.0001 (0.0001)
					*p* = 1	*p* = 1	*p* = 1
	**Alleles**	**T**	304 (97.5)	322 (98.2)	–	–	–
		C	8 (2.5)	X	–	–	–

Model 1: adjusted for age and sex

Model 2: additionally adjusted for Adiponectin, cortisol

We investigated whether the SNPs of the *ADIPOQ* gene affected the plasma levels of adiponectin and cortisol. There was no significant difference in adiponectin levels between the carriers of the four different genotypes at the locus mentioned above. There was no significant relationship between the *ADIPOQ* polymorphisms and cortisol levels, except between rs1501299 polymorphism and cortisol in obese individuals (0.039) (Table 4).

**Table 4:** Serum Adiponectin and cortisol levels in subjects with different genotypes of ADIPOQ SNPs

	Genotypes	Adiponectin			cortisol		
		BMI < 25	BMI ≥ 25	*P*-value	BMI < 25	BMI ≥ 25	*P*-value
rs17300539	GG	8.30 (4.95)	7.01 (4.78)	N.S^a,b^	11.53 (8.45)	9.62 (7.16)	N.S^a,b^
	GA	8.54 (4.55)	7.68 (6.50)		13.65 (10.01)	9.36 (9.95)	
	AA	9.4			8.4		
rs266729	CC	8.41 (4.58)	6.94 (4.21)	N.S^a,b^	11.74 (8.35)	9.53 (7.08	N.S^a,b^
	GC	8.744 (5.32)	7.58 (5.78)		12.28 (8.18)	9.53 (6.35)	
	GG	9.84 (4.30)	7.53 (7.64)		13.9 (6.36)	11.77 (9.58)	
rs1501299	GG	9.34 (5.15)	7.17 (4.60)	N.S^a,b^	12.17 (7.99)	11.12 (7.50)	N.S^a^ 0.039^b^
	GT	8.05 (3.88)	7.45 (5.68)		11.53 (8.45)	8.14 (6.04)	
	TT	7.65 (3.40)	8.26 (3.74)		11.74 (8.35)	10.82 (6.34)	
rs17366743	CC	18.10		0.071^a^ 0.095^b^	13.70		N.S^a,b^
	TC	11/8 (6.98)	10.52 (7.78)		13.15 (5.02)	13.17 (5.97)	
	TT	8.13 (4.69)	6.90 (4.77)		11.72 (8.80)	9.40 (7.53)	

^a^Comparison for BMI < 25

^b^Comparison for BMI ≥ 25

Difference in adiponectin and cortisol levels between the genotypes groups for ADIPOQ SNPs, analyzed by ANOVA for normally distributed data and kruskal-Wallis test for without normal distribution

The Spearman correlation and Pearson correlation analysis were performed to find out the correlation of serum adiponectin levels with biochemical and clinical variables. According to Table 5, Adiponectin level was positively correlated with HDL-C, and negative or inverse correlations were found between Adiponectin and each BMI, waist circumference(WC), HIP, Waist to hip, TG, TC, SBP, DBP, and FBS (Table 5).

**Table 5:** Correlations of the serum adiponectin level with different parameters in participants

Parameters	Correlation Coefficient	***P***-value
Age	0.095	0/057
Body mass index	−0.147	0.003
Weight	−0.360	0.002
Height	−0.213	0/000
Hip circumference	−0.179	0/002
Waist circumference	− 0.324	0/000
Waist to hip	−0.371	0/000
Triglyceride	−0.277	0/000
Total cholesterol	−0.041	0/000
High density lipoprotein cholesterol	0.295	0/000
Low density lipoprotein cholesterol	−0.014	0/801
Systolic blood pressure	−0.134	0/019
Diastolic blood pressure	−0.149	0/009
Cortisol	−0.038	0/890
FBS	−0.299	0/000

The Spearman correlation and Pearson between adiponectin level with biochemical and clinical variables

Haplotype analyses: The haplotypes frequencies of the *ADIPOQ* SNPs (rs1501299, rs266729, rs17300539, and rs17366743) showed that the GGGGCCTT haplotype (26.9%) had the highest frequency. No significant association was found between the haplotypes in *ADIPOQ* and obesity, Adiponectin, and cortisol. (Table is not shown).

## Discussion

In the present study, significant differences in adiponectin and cortisol levels were observed in the two groups (BMI < 25, BMI ≥ 25) and in other biochemical and anthropometric factors, including waist circumference, hip circumference, waist to hip ratio, triglyceride, LDL-C, blood pressure, and cortisol; however, there was no significant difference in HDL-C between the case and control groups. In many studies, lower adiponectin levels and higher cortisol levels were reported in obesity, while our findings demonstrated a fall in cortisol level in obese people [24, 25]. A metabolic disorder is often linked with cortisol plasma level fluctuation [26]. In Canada, the population cortisol levels were not different between BMI categories, but the level of Adiponectin was different in BMI categories, and adiponectin level was lower in obese individuals [27]. The current findings showed significant differences in BMI, glucose, total cholesterol, LDL, triglycerides, and HDL between the two groups, which were similar to the studies on patients with type 2 diabetes mellitus in a Chinese population [28] and on people with severe obesity in Italy [25]. In the North Indian Punjabi population, the results agreed with our findings; the obese subjects had significantly higher mean values for BMI, WC, WHR, WHtR, SBP, DBP, fasting glucose level, TC, and LDL-C compared to the nonobese, but there were no significant differences in HDL-C, TG [29]. By increasing the transcription of genes involved in fatty acid metabolism, including peroxisome proliferator-activated receptor-α, Adiponectin activates PPAR-α which leads to higher levels of molecules involved in fatty acid transport protein, energy dissipation such as CD36, and uncoupling protein-2, as they can elevate fatty acid oxidation. The rates of energy expenditure and fat oxidation play a role in determining body weight [3]. Therefore, Adiponectin was indicated to prevent the accumulation of lipids in insulin target tissues by stimulating the oxidation of fatty acids. Our results agree with the Taiwanese population’s results that adiponectin expression in obese subjects was lower than in the nonobese. Also, a significant decrease was observed in plasma adiponectin level in the subjects with increased obesity [30] and MetS components [31]. In a Japanese population, adiponectin levels were strongly and inversely associated with the risk of type 2 diabetes [32].

Adipokine genes do not directly cause disease but can enhance the effect of environmental factors [28]. Single-nucleotide polymorphisms (SNPs) and haplotypes of adiponectin gene are associated with obesity [25], but in this study, no significant association was found between any of SNPs *ADIPOQ* (rs1501299, rs266729, rs17300539, and rs17366743) with BMI under the recessive, dominant and additive models. A study of four polymorphisms (rs1501299, rs266729, rs17300539, and rs17366743) in an Italian population showed that rs17300539 and rs1501299 were associated with severe obesity [25]. The rs17366743 SNP was associated with diabetes incidence in European descent of Boston [19]; on the other hand, when metabolic syndrome traits were analyzed in association with rs17300539, none showed a significant association with this variant [12]. Investigation of SNPs rs17300539, and rs266729 withT2DM in Tunisian Arabs, using additive, dominant, and recessive genetic models showed that the two SNPs were significantly associated with T2DM [6]. In Oman, obesity was associated with the *ADIPOQ* SNP rs266729 but not with rs17300539, and in young Croatians, SNP rs266729 showed an increased risk for central obesity. SNP rs266729 was not associated with obesity in French, Italian and Chinese populations, which were in agreement with our findings. Evidence was observed for the association of SNP rs266729 with obesity in the Arab population [12], but other populations reported varying results of the association between rs266729 and the risk of obesity or MetS [33]. The rs266729 was associated with T2DM in the French and Swedish [3]. In young Nigerian adults, the rs266729 was associated with increased obesity using three genetic models: additive, codominant, and recessive [34], and there were associations of central obesity with rs266729 in a population from China [28]. The rs266729 was associated with T2DM in Japanese subjects [3]. The rs1501299 polymorphism significantly increased the risk of obesity and MetS in the North Indian Punjabi population. TT Genotype of rs1501299 (+276G > T) polymorphism had a significantly higher risk of obesity, and the GG and GT genotypes frequencies were higher in nonobese subjects but not statistically significant [29]. Rs1501299 was not associated with type 2 diabetes risk under two genetic models (general or additive) [32]. In the Italian population [25], China [28], and young Nigerian adults [34], the rs1501299 was associated with obesity. In addition, *ADIPOQ* SNP rs1501299 was associated with CAD risk in Iranian subjects with T2DM [35]. A Study in an Italian population demonstrated that rs17366743 was not associated with severe obesity [25] but was associated with diabetes in European descents of Boston [19]. The results reported on SNPs have differed in other populations and studies. Expectedly, there may be variability in SNP distribution in different populations, even within the same ethnicity [34]. No significant associations of rs17300539 SNP with adiponectin levels were observed in Russian Federation [36]. In obese Japanese subjects [25] and European descents of Boston and obese Portuguese pediatric population, rs17300539 was associated with plasma adiponectin level (19, 25). In a study conducted in Italy [37], adiponectin levels were influenced by rs17300539. Adiponectin level was lower in GG genotype than individuals carrying G/A [37, 38]. In Italy and the Japanese populations, a decrease in adiponectin level was observed for G carriers of rs266729 compared with the non-carriers [37, 39]. However, the subjects with the GG genotype of rs266729 had lower adiponectin levels than carriers among people of the Russian Federation [36]. The studies have indicated that the G allele of rs266729 modifies the sequence for one of the transcriptional regulatory protein binding sites and decreases adiponectin promoter activity [40, 41]. Lower serum adiponectin was associated with rs266729 in French and Amish subjects [3]. Adiponectin level was higher in GG subjects of rs266729 in participants from London hospitals [17]. GG genotype of *ADIPOQ* rs266729 was associated with participants with lower BMI in China and with obesity, higher waist circumference, BMI in Oman [12] and FBS in Italy [37]. A study in China [42] and European descent of Boston [19] showed no significant association between rs266729 and adiponectin level. Many studies demonstrated the association of SNP rs1501299 in adiponectin gene with adiponectin levels [19, 25], but no association was found between rs1501299 and adiponectin levels in Italy [4] and Iran [35]. Studies have shown that SNP rs1501299 is associated with higher serum levels of Adiponectin [43], and it is a protective factor for diabetes Mellitus, hypertension, coronary artery disease, and dyslipidemia in American, Japanese, Finnish [35], and Korean populations [35, 39]. No significant association was found between rs17366743 SNP and adiponectin concentration in Russian Federation [36]. The different association between SNPs and adiponectin levels may be due to the level of obesity of the studied population [19]. No association has been observed in several genome-wide scans between the adiponectin level and ADIOPQ polymorphisms [4, 19, 35, 42]. In this regard, any possible association has been due to a linkage between SNPs adiponectin and another mutation in the other genes close to the *ADIPOQ* gene [19, 25, 35]. In general, different results in the relationship of genetic variants of *ADIPOQ* with obesity and metabolic syndrome can be due to differences in ethnic populations, communication methods, and the study power [12]. The different association results of SNPs adiponectin with obesity and obesity-related diseases are not unexpected, according to reported ethnic and geographical differences in the adiponectin gene [7]. SNPs (rs17300539, rs266729, rs1501299, rs17366743) were not associated with adiponectin levels in the present study. The lack of associations may be due to the insufficient power of our study, in which the number of participants was limited.

Regarding adiponectin correlation with other parameters, there was a negative correlation between Adiponectin with BMI, WC, HIP, Waist to hip, TG, TC, SBP, DBP, and FBS, and positive correlations between adiponectin and HDL cholesterol. The negative association between BMI and adiponectin levels in the Canadian population is similar to that in our findings [27]. The studies have shown a negative relationship between Adiponectin with triglyceride (TG) levels [37, 44], BMI [45, 46], Metabolic syndrome [47] and a positive relationship between adiponectin and high-density lipoprotein (HDL)-cholesterol in obese subjects [37]. Positive correlations between Adiponectin and HDL cholesterol support the thesis that Adiponectin is involved in regulating cholesterol, and it seems that HDL-C exerts reciprocal effects on adiponectin expression in 3 T3-L1 and adipocyte metabolism. HDL-C enhances adiponectin expression in adipose tissue cells in a phosphatidylinositol-3-kinase (PI3K) dependent manner, and Plasma adiponectin concentrations are elevated. Croatia authors have not observed a correlation between Adiponectin and LDL-C and TC, which contradicts our findings, whereas some studies showed a significant association between adiponectin and lipid profile [15]. There is scanty and inconsistent research on the relationship between Adiponectin and cortisol. For example, research by Gavrila has shown that cortisol has an inhibitory effect on Adiponectin [48], whereas Demir showed a positive correlation between serum adiponectin level and cortisol [49]. Other studies agree that there was no correlation between adiponectin levels and cortisol [50]. The limitation of this study is that there was only one sampling procedure, whereas the level of cortisol obtained may change at different time points, so that a normal sampling procedure could cover an average value of several daily measurements.

## Conclusion

In the present study, no associations were observed between SNPs(rs1501299, rs266729, rs17300539, and rs17366743 or haplotypes with obesity. Adiponectin and cortisol were associated with obesity. Finally, rs1501299 was associated with cortisol in subjects with BMI ≥ 25.

## Data Availability

Data supporting this study’s findings are available from the corresponding author on request.

## Abbreviations

BMI: Body mass index
TLGS: Tehran Lipid and Glucose Study
PCR-RFLP: Polymerase chain reaction-restriction fragment length polymorphism
SNPs: Single nucleotide polymorphisms
LDL-C: Low-density lipoprotein cholesterol
AMPK: AMP-dependent kinase
WC: Waist circumference
HC: Hip circumference
TG: Triglyceride
TC: Total cholesterol
SBP: Systolic blood pressure
DBP: Diastolic blood pressure
FBG: Fasting blood glucose
HDL-C: High-density lipoprotein cholesterol
